# How LGBT+ Young People Use the Internet in Relation to Their Mental Health and Envisage the Use of e-Therapy: Exploratory Study

**DOI:** 10.2196/11249

**Published:** 2018-12-21

**Authors:** Mathijs Lucassen, Rajvinder Samra, Ioanna Iacovides, Theresa Fleming, Matthew Shepherd, Karolina Stasiak, Louise Wallace

**Affiliations:** 1 School of Health, Wellbeing and Social Care The Open University Milton Keynes United Kingdom; 2 Faculty of Sciences University of York York United Kingdom; 3 Faculty of Health Victoria University of Wellington Wellington New Zealand; 4 Faculty of Education and Social Work University of Auckland Auckland New Zealand; 5 Faculty of Medical and Health Sciences University of Auckland Auckland New Zealand

**Keywords:** sexuality, LGBT, transgender, depression, adolescent, psychotherapy, mental health, computer games, computerized CBT, e-therapy

## Abstract

**Background:**

Lesbian, gay, bisexual, and transgender (LGBT) youth and other young people diverse in terms of their sexuality and gender (LGBT+) are at an elevated risk of mental health problems such as depression. Factors such as isolation and stigma mean that accessing mental health services can be particularly challenging for LGBT+ young people, and previous studies have highlighted that many prefer to access psychological support on the Web. Research from New Zealand has demonstrated promising effectiveness and acceptability for an LGBT+ focused, serious game–based, computerized cognitive behavioral therapy program, Rainbow Smart, Positive, Active, Realistic, X-factor thoughts (SPARX). However, there has been limited research conducted in the area of electronic therapy (e-therapy) for LGBT+ people.

**Objective:**

This study aimed to explore how and why LGBT+ young people use the internet to support their mental health. This study also sought to explore LGBT+ young people’s and professionals’ views about e-therapies, drawing on the example of Rainbow SPARX.

**Methods:**

A total of 3 focus groups and 5 semistructured interviews were conducted with 21 LGBT+ young people (aged 15-22 years) and 6 professionals (4 health and social care practitioners and 2 National Health Service commissioners) in England and Wales. A general inductive approach was used to analyze data.

**Results:**

LGBT+ youth participants considered that the use of the internet was ubiquitous, and it was valuable for support and information. However, they also thought that internet use could be problematic, and they highlighted certain internet safety and personal security considerations. They drew on a range of gaming experiences and expectations to inform their feedback about Rainbow SPARX. Their responses focused on the need for this e-therapy program to be updated and refined. LGBT+ young people experienced challenges related to stigma and mistreatment, and they suggested that strategies addressing their common challenges should be included in e-therapy content. Professional study participants also emphasized the need to update and refine Rainbow SPARX. Moreover, professionals highlighted some of the issues associated with e-therapies needing to demonstrate effectiveness and challenges associated with health service commissioning processes.

**Conclusions:**

LGBT+ young people use the internet to obtain support and access information, including information related to their mental health. They are interested in LGBT-specific e-therapies; however, these must be in a contemporary format, engaging, and adequately acknowledge the experiences of LGBT+ young people.

## Introduction

### The Mental Health of Lesbian, Gay, Bisexual, and Transgender Young People

Lesbian, gay, bisexual, and transgender (LGBT) young people and other young people diverse in terms of their sexuality and gender (LGBT+) are thought to form up to 12% of the adolescent population [1,2]. Recent systematic reviews have indicated that LGBT+ young people are more likely to experience mental health problems such as depression, self-harm, and suicidality than their age-matched peers [1,3]. For instance, in a meta-analysis of population-based studies, lesbian, gay, bisexual, and other sexuality diverse youth were almost 3 times more likely to have depressive symptoms or a depressive disorder in comparison with heterosexual youth [1]. These greater mental health risks are hypothesized to be caused by minority stress, whereby it is mistreatment and high levels of stress that places LGBT+ young people at greater risk [4,5]. In particular, experiencing mistreatment and stress results in LGBT+ individuals frequently internalizing the negativity associated with anti-LGBT+ messages. This, in turn, can lead to self-loathing and a range of unhelpful cognitions, which are then thought to place LGBT+ young people at greater risk of mental health problems, such as depression [6]. Moreover, it is not unusual for LGBT+ young people to face the challenge of navigating multiple stigmas related to differences such as being LGBT+ and having mental health problems [7] or being an ethnic minority and LGBT+ [8]. In addition to being different and having greater mental health needs, LGBT+ youth are frequently required to manage antagonistic environments in an ongoing manner while simultaneously struggling with a general lack of social support [7]. Given these challenges, it would be logical to assume that there has been a strong focus on providing psychotherapeutic supports for LGBT+ young people. Unfortunately, to date, this has not been the case. Research in the area of psychotherapeutic interventions for this unique population is limited, and LGBT+ young people report difficulties accessing face-to-face professional help for their emotional concerns [9].

### Lesbian, Gay, Bisexual, and Transgender Young People and the Internet

The internet has opened up a range of possibilities for LGBT+ young people, including psychosocial support and self-care for mental health problems. This assistance may be especially pertinent for LGBT+ young people, where parental support, a crucial protective factor in adolescence, may be lacking [10]. For example, LGBT+ young people can readily connect with others on the Web, irrespective of where they reside, and as such, the internet has become an important source of support, information, and connection [11,12]. LGBT+ young people can also obtain informal help on the Web to assist them in managing LGBT+ specific mistreatment (such as homophobia) and in coping with emotional distress [7].

To date, little has been published on how LGBT+ young people use the internet to successfully support their mental health. However, research from Canada and the United States has highlighted that LGBT+ young people are particularly active internet users [13] and use a wide range of Web-based media for information, resources, and support [13,14]. Work from Australia has also highlighted the relative importance of social media for LGBT+ young people, especially for transgender young people [15]. For example, 75% of transgender young people in a study by Strauss et al (n=711) reported that social media use was the most common Web activity participants engaged in to help them feel better [15]. In England, McDermott et al have conducted a Department of Health–commissioned mixed-methods study focused on suicidality, self-harm, and help-seeking in LGBT+ young people [16]. Most LGBT+ young people in their study reported a preference for accessing help through the internet, followed by face-to-face and then text messaging forms of support [16]. Young people in their study had the most positive experiences when asking for help on the Web, as well as from friends, or from LGBT+ youth groups. In contrast, primary care general practitioners and mental health services delivered by the National Health Service (NHS) received low ratings in terms of their perceived helpfulness by LGBT+ young people with mental health problems [16]. Although McDermott et al found that LGBT+ young people value internet-based supports, little detail about the types of e-therapy or on the Web help that could be provided were outlined. Instead, they advocated for “...a more imaginative approach to providing support and help” (p. 170) for LGBT+ young people, and they suggested that services be situated in nonclinical settings such as online [17].

### Lesbian, Gay, Bisexual, and Transgender Young People and Electronic Therapy

Formal online mental health interventions have become far more accessible in the last decade; in particular, in the form of computerized cognitive behavioral therapy (cCBT), which has become an effective and recommended form of e-therapy for the treatment of depression [18]. As an intervention, cCBT is particularly promising for LGBT+ young people as it offers opportunities to increase access to treatment. This is because it does not necessarily require therapist support; it can be made freely available to end users; it can be completed in privacy; and it can be made accessible to socially and geographically isolated individuals via the internet [6].

Although LGBT+ young people are an underserved population in terms of their mental health needs, few therapies, online [19] or offline, have been developed for them. A recent systematic review of psychosocial interventions for mental health problems among LGBT+ young people stated that only “A few promising psychological therapies adapted to meet the needs of LGBTQIA [Lesbian, Gay, Bisexual, Transgender, Queer, Intersex, and Asexual] individuals have emerged in recent years” (p. 2) [10]. This review from Van Der Pol-Harney and McAloon [10] and another review, conducted by Hobaica et al [20], identified only 1 computerized intervention to support the mental health of LGBT+ young people, which was not focused on addressing drug usage, specifically the program Rainbow Smart, Positive, Active, Realistic, X-factor thoughts (SPARX) [10,20]. This intervention is a 7-module cCBT program created for sexual minority young people in New Zealand [21] and is delivered in the English language. Rainbow SPARX has been evaluated in an open trial with LGBT+ young people [21,22] but to date has not been trialed nor appraised for use in the United Kingdom or anywhere other than New Zealand.

It is perhaps surprising that, to date, no e-therapies have been developed or tested for LGBT+ young people in the United Kingdom. This is particularly noteworthy, given that LGBT+ young people have indicated a preference for Web-based help, and there is a strong policy push toward providing more e-therapies in the United Kingdom. For instance, one of the key recommendations when planning services from 2016 to 2021 from the Mental Health Taskforce to the NHS in England was that the NHS “...should expand work on NHS Choices [the main patient-facing website] to raise awareness and direct people to effective digital mental health products...” (p. 42) [23]. Using e-therapies that have already been developed and tested elsewhere could enable health services in the United Kingdom to provide a range of effective treatment options, including for LGBT+ young people.

### This Study

**Figure 1:**
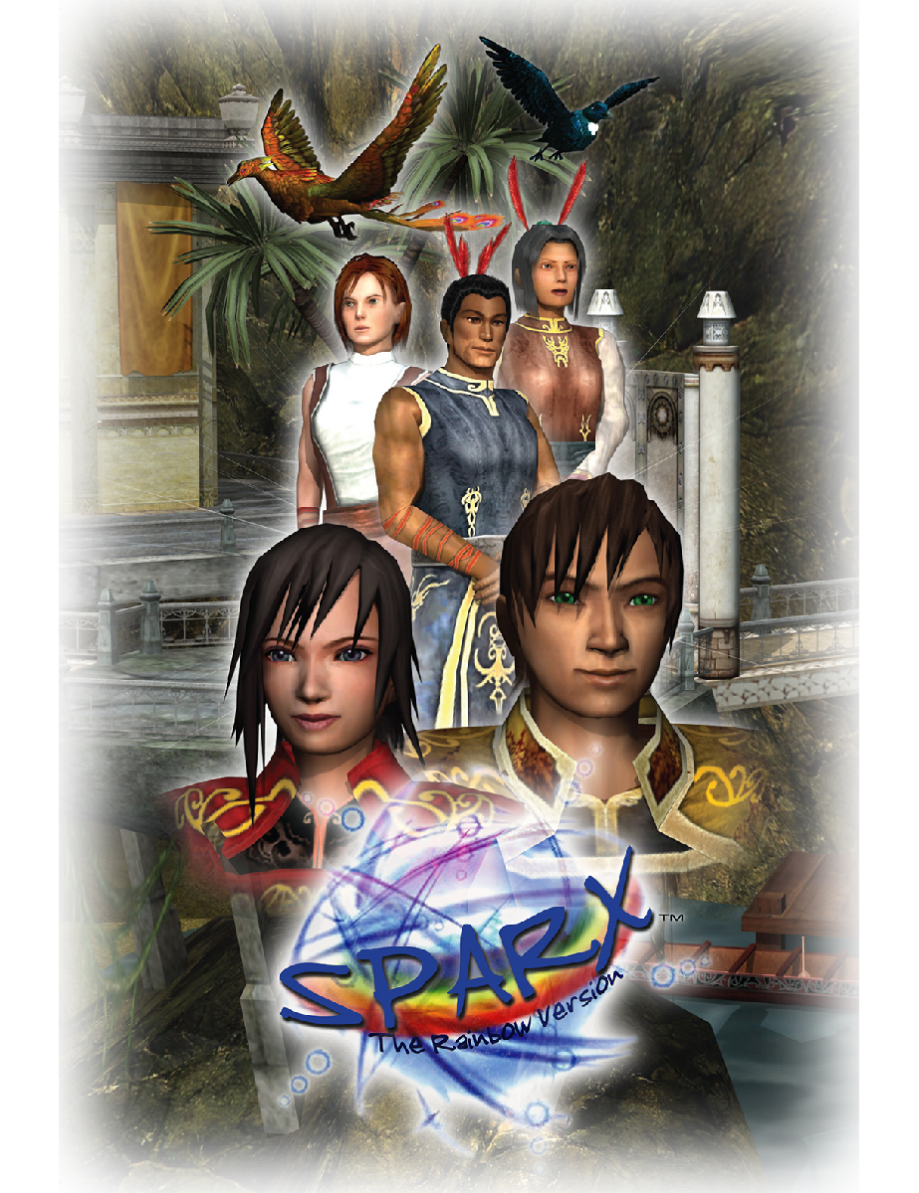
Rainbow SPARX image. SPARX: Smart, Positive, Active, Realistic, X-factor thoughts.

This study sought to explore the acceptability of a non-United Kingdom-developed intervention, Rainbow SPARX (Figure 1), for use in British settings. At present, Rainbow SPARX is the only e-therapy focused on addressing depression in LGBT+ youth. Rainbow SPARX is an adapted version of SPARX [21], which is a serious game and form of cCBT for the treatment of depressive symptoms. It uses the medium of a fantasy world, where the user’s avatar is faced with a series of challenges to rid a virtual world of gloom and negativity [21,24]. SPARX uses computer graphics and interactive exercises to engage users. Each of the program’s 7 modules takes approximately 30 min to complete, and modules have a direct teaching component where the skills from the fantasy world are applied to real life [21]. A randomized controlled trial (RCT) of SPARX was conducted in New Zealand in a general population of young people seeking help for their mental health issues [24]. Per-protocol analyses (n=143) showed that SPARX was not inferior to treatment-as-usual, and postintervention results showed a mean reduction of depressive symptoms [24]. SPARX is now freely available on the Web to anyone with a New Zealand internet protocol address. Following feedback from LGBT+ young people about the importance of refining SPARX for this population, Rainbow SPARX (or SPARX: The Rainbow Version) was made in consultation with LGBT+ young people [6]. An open trial of Rainbow SPARX (n=21) was conducted with LGBT+ young people in New Zealand [21]. This preliminary research highlighted that Rainbow SPARX was a promising intervention in terms of effectiveness by showing a significant reduction of depressive symptoms. This research also showed that it was deemed by participants to be an acceptable form of therapy and was judged to be feasible to deliver [21,22]. However, the cultural relevance of an e-therapy designed in New Zealand needs to be examined in terms of its suitability to young people in a different cultural context. This is because mental health interventions frequently need to be modified to best meet their users’ needs across cultural contexts [25,26], and SPARX includes various Māori (ie, indigenous New Zealand) and other South Pacific references. Furthermore, the acceptability of the program to professionals (including mental health practitioners) and commissioners is important to assess, as their views are key to ensuring the funding and promotion of e-therapies is achieved within health services.

Building on prior mixed-methods research related to Rainbow SPARX [6,21,22,27], in this study, we had 2 key research objectives: (1) to explore how and why LGBT+ young people use the internet to support their mental health and (2) to consider the extent to which LGBT+ young people, their parents (or guardians), and professionals think an e-therapy, such as Rainbow SPARX, could be a useful tool to assist in supporting the mental health of LGBT+ young people.

The consolidated criteria for reporting qualitative research [28] were used to guide reporting in this study.

## Methods

Ethical approval for this study was obtained from The Open University’s Human Research Ethics Committee (reference HREC/2017/2507/Lucassen/1).

### Participants

For inclusion in this study, participants needed to be living in the United Kingdom and to be:

- An LGBT+ young person aged 12 to 22 years,
- The parent or guardian of an LGBT+ young person, or
- A professional with either expertise in working with LGBT+ young people or in adolescent mental health service provision or commissioning.

Young people who were exclusively heterosexual and cisgender (ie, those who experience congruence between their gender identity and the sex they were assigned at birth) were not eligible to participate.

### Recruitment

Recruitment of LGBT+ young people in research projects is often fraught with challenges and ethical issues [6,27]. For example, many LGBT+ young people are not *out* to their parents, and for those aged less than 16 years, written parental consent is almost always required if they wish to participate in mental health–related studies. In light of the challenges associated with conducting research in this field, we took a pragmatic approach to recruitment in 2 key ways. First, the age inclusion criterion for young people was initially set to be up to 19 years, but because there was considerable interest from LGBT+ young adults (who were already existing members of the youth groups involved in this study), the age range was extended. Hence, participants in this study included young people who were existing members of an LGBT+ youth group. Second, 1 young person who was not a member of any LGBT+ youth groups, but was very keen to take part in the study, was offered an individual interview (with ML and RS present).

Potential participants who were LGBT+ young people and parents of LGBT+ young people were informed about the study via advertising on social media (eg, on closed Facebook groups for LGBT+ individuals and their allies) and from networks of LGBT+ organizations known to or recommended to the authors. For instance, key LGBT+ youth organizations with a Web presence in major cities in the United Kingdom were contacted in London, Cardiff, Manchester, Edinburgh, and Belfast, and staff in these organizations were informed about this research. Primarily, young people heard about the study from staff supportive of this project at relevant LGBT+ youth groups. Potential participants who were professionals were known to the authors and were approached by either ML or LW.

### Procedures

The interviews and focus groups were led by ML (a gay and queer male-identified academic experienced in youth mental health work) with assistance from fellow academics (ie, RS and LW). Written parental and participant informed consent was obtained from those LGBT+ young people aged between 12 and 15 years. Adult participants and LGBT+ young people aged 16 years or older provided written consent for themselves. Each interview and focus group began with personal introductions (eg, names and correct gender pronouns) and confirmation of the research objectives and processes; ML explained that he led the development of Rainbow SPARX and also highlighted his interest in supporting the mental health of LGBT+ young people. The semistructured interview guide used in the focus groups and interviews was developed by the authors and reviewed by colleagues independent of this study. See Multimedia Appendix 1 for the focus group guide (the focus group questions were adjusted for the interview format). The questions were open-ended (eg, *What are the main reasons why LGBT+ young people use the internet?* and *In what ways should Rainbow SPARX be adapted to meet the needs of LGBT+ young people in the United Kingdom?*), and discussion was actively encouraged. During the interview or focus group, participants were shown module 1 of Rainbow SPARX, with a single participant controlling the game at any one time while others watched and commented. Participants did not need to have any prior knowledge of this program, serious gaming, or e-therapy. Young people were offered a £20 gift voucher as a gratuity. Interviews and focus groups were digitally audio-recorded and professionally transcribed. The transcripts were thoroughly checked against the digital recordings by ML for accuracy before data analysis began.

Participants completed a brief demographics questionnaire at the end of the interview or focus group. Specifically, young people were asked questions, which included open-response items asking their age, gender or gender identity, and ethnicity. They were also asked about their sexuality (ie, *Please circle below which applies for you: Lesbian, Gay, Bisexual, Questioning, Queer, Not heterosexual, Other [for other, please explain...]*), a closed question about whether they would use Rainbow SPARX and another closed question about whether or not they had suffered from feeling down or low for more than a few days. Professionals were asked their designation (ie, *Please circle below which applies for you: Mental health professional, Commissioner, LGBT Stakeholder, Other* *[for other, please explain...]*), gender or gender identity, ethnicity, and if they would recommend the use of Rainbow SPARX. Despite considerable attempts to recruit parents of LGBT+ young people, none enrolled in this study (ie, no parents completed the brief questionnaire or participated in an interview or focus group).

In total, 3 focus groups (which were young people and the LGBT+ staff members responsible for these groups only) and 5 interviews (1 LGBT+ young person interview and 4 interviews with professionals) occurred during 2017. They lasted between 51 min and 1 hour and 24 min (mean length 62 min).

### Data Analysis

We used a general inductive approach for data analysis [29,30]. This approach focuses on eliciting views and perspectives of participants using preexisting research objectives, rather than generating new theories. A thematic analysis was used because the aim was to explore common themes and interrelationships between themes [30]. Focus group and interview transcripts were read and reread with the research objectives in mind by ML, RS, and II. Initial codes were independently developed by ML, RS, and II for a focus group and 2 interviews, and after discussion, a common coding framework was developed. This framework was applied by ML and RS to all interviews and focus groups. Codes were then reviewed for redundancies and overlap before higher-order units were created and clustered together. Themes and subthemes were developed and agreed on in consultation with all authors. Microsoft Excel 2013 was used to manage the data and support analyses.

## Results

### Participants

A total of 21 LGBT+ youth participants took part and they were aged between 15 and 22 years (mean age 17.9 years) (see Tables 1 and 2 for details). Moreover, 6 health and social care professionals participated: 2 were NHS commissioners (1 managerial and 1 clinical), 3 professionals had expertise in LGBT+ youth mental health, and 1 participant was experienced at developing e-therapies.

**Figure 2:**
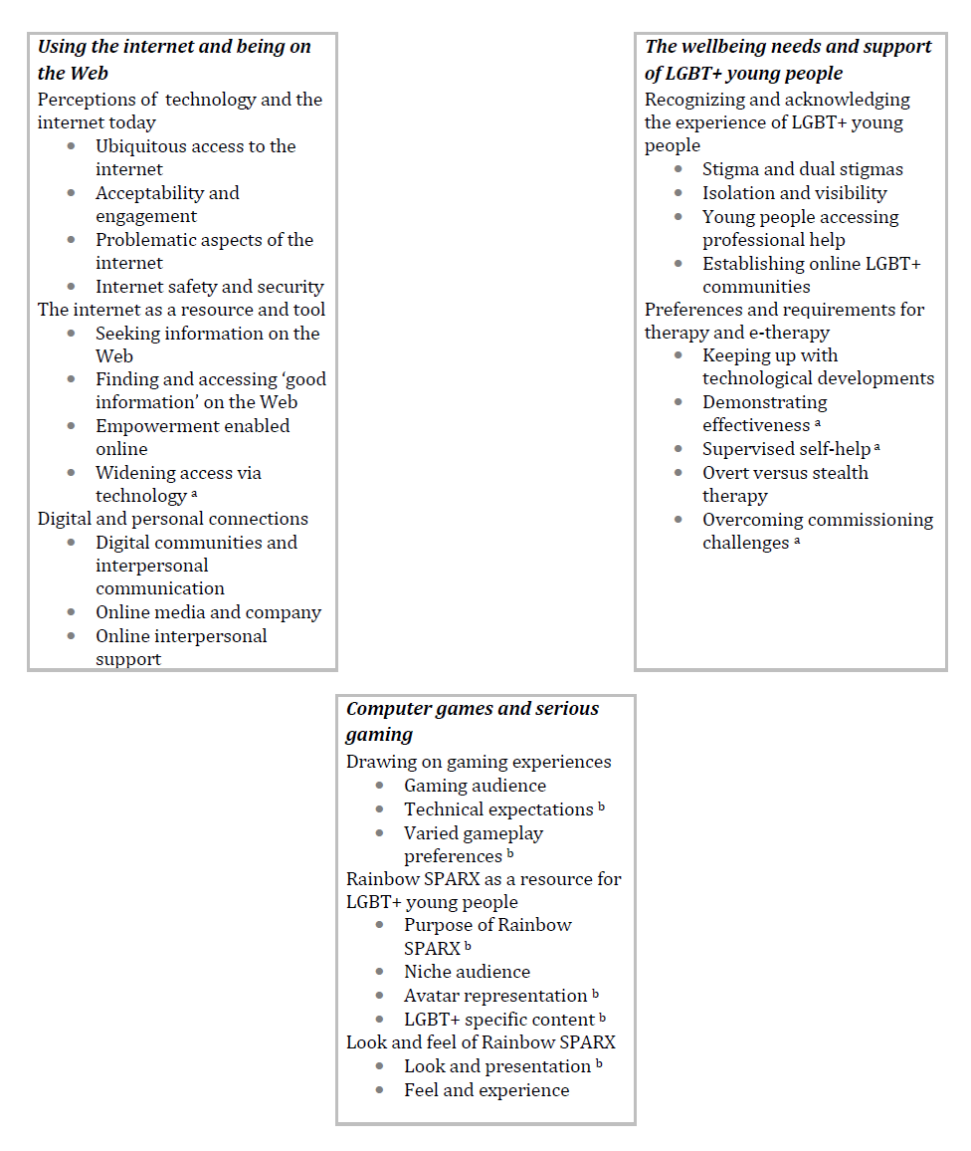
Overview of results. the letter "a" indicates subthemes of particular salience to professionals, and "b" indicates subthemes of particular salience to LGBT+ young people. LGBT: lesbian, gay, bisexual, and transgender; SPARX: Smart, Positive, Active, Realistic, X-Factor Thoughts.

Figure 2 provides an overview of the study’s results. There were 3 main overarching domains: specifically, using the internet and being on the Web, computer games and serious gaming, and the well-being needs and support of LGBT+ young people. Each domain included 2 or 3 themes and then several associated subthemes.

### Using the Internet and Being On the Web

The domain of *using the internet and being on the Web* consisted of 3 themes: perceptions of technology and the internet today; the internet as a resource and tool; and digital and personal connections.

#### Perceptions of Technology and the Internet Today

Several LGBT+ young people and professionals highlighted that the internet is ubiquitous and influential:

I think we have grown up and been shaped by the internet, so we know it intrinsically in a way that perhaps older generations don’t...

YP16, young person, focus group 2

It’s fast; it’s there; they [LGBT+ young people] have access to [the]internet practically everywhere.

P5, professional, interview 4

In contrast, the minority of young people who do not have ready access to the internet were viewed by participants as being socially excluded.

It would appear that from an early age, youth participants begin to discern Web-based resources in terms of their acceptability, and this is then used to assess the suitability of Web-based solutions for different aspects of their lives:

...there’s online counselling or online suicide helplines, so if you’re feeling suicidal or depressed or whatever or you’re going to relapse on drugs or self-harm then you can have like this, either you can call a number, you can find that on the internet, or you can do an online chat, which I think is really good, because most people, calling someone makes you feel quite anxious and stuff and I think a lot of people don’t want to call, because it’s a lot of effort.

YP20, young person, focus group 3

Web-based environments were viewed as being unhealthy or unhelpful in several ways by a range of participants. For instance, LGBT+ youth participants cited *Pro-ana* and self-harm sites that *glamorize* mental health problems, such as anorexia and self-harming, as problematic. Professionals also described ways in which the internet creates difficulties for young people in either a more general sense (eg, by young people spending too much time on the Web, and hence, avoiding the *real world*) or in very specific ways. For example, considering how Web-based pornography can generate issues for LGBT+ young people:

[it] can maybe make people feel less confident about their bodies, less confident about their sex and their relationships as a result...

P3, professional, interview 2

**Table 1:** Participants’ demographic information (grouped by interviews).

Format (researchers present) [setting^a^]	Participant number	Participant category	Age (years)	Sexuality	Gender/Gender identity	Ethnicity
Group interview 1 (ML and LW) [participant’s workplace]	P1	Professional (commissioner)	—^b^	—	Male	White British
Group interview 1 (ML and LW) [participant’s workplace]	P2	Professional (commissioner)	—	—	Female	White Other
Individual interview 2 (ML) [participant’s workplace]	P3	Professional (LGBT^c^+ stakeholder)	—	—	Male	White British
Individual interview 3 (ML) [participant’s workplace]	P4	Professional (mental health)	—	—	Male	White British
Individual interview 4 (ML) [participant’s workplace]	P5	Professional^d^ (LGBT+ stakeholder)	—	—	Male	White British
Individual interview 5 (ML and RS) [city library]	YP12	Young person	19	Gay	Male	White British

^a^Interviews and focus groups were conducted in private spaces.

^b^Not asked.

^c^LGBT: lesbian, gay, bisexual, and transgender.

^d^This professional attended 2 focus groups and participated in an individual interview.

**Table 2:** Participants’ demographic information (grouped by focus groups).

Format (researchers present) [setting^a^] and participant number		Participant category	Age (years)	Sexuality	Gender/Gender identity	Ethnicity
**Focus group 1 (ML) [LGBT^b^+ youth center]**							
	P5	Professional^c^ (LGBT+ stakeholder)	—^d^	—	Male	White British
	P6	Professional^e^ (mental health)	—	—	Female	Black British
	YP1	Young person	18	Gay	Male	White British
	YP2	Young person	19	Not heterosexual	Male	White British
	YP3	Young person	19	Questioning	Male	White British
	YP4	Young person	21	Queer	N/A^f^	White Irish
	YP5	Young person	18	Gay	Male	White British
	YP6	Young person	21	Queer	Nonbinary	White British
	YP7	Young person	22	Pansexual	FTM^g^	White British
	YP8	Young person	16	Gay	Male	Mixed
	YP9	Young person	18	Gay	Male	White British
	YP10	Young person	18	Bisexual	Female	White British
	YP11	Young person	19	Gay	Male	White British
**Focus group 2 (ML) [community center]**						
	YP13	Young person	17	Bisexual and questioning	Slightly queer	Caucasion (sic)
	YP14	Young person	20	Lesbian	Female	White Welsh
	YP15	Young person	15	Not heterosexual and asexual	Trans female	Caucasian
	YP16	Young person	17	Queer	Queer	White British
	YP17	Young person	17	Pansexual	Woman	White Welsh
	YP18	Young person	16	Lesbian	Female	White British or Welsh
**Focus group 3 (ML) [LGBT+ youth center]**						
	P5	Professional^d^ (LGBT+ stakeholder)	—	—	Male	White British
	P6	Professional^e^ (mental health)	—	—	Female	Black British
	YP19	Young person	15	Transgender or pansexual	FTM transgender	White British
	YP20	Young person	15	Gay, questioning, queer, and not hetero	Male cisgender	White British
	YP21	Young person	15	Gay, questioning, queer, and not hetero	Male	Black British

^a^Interviews and focus groups were conducted in private spaces.

^b^LGBT: lesbian, gay, bisexual, and transgender.

^c^This professional attended 2 focus groups and participated in an individual interview.

^d^Not asked.

^e^This professional attended 2 focus groups.

^f^N/A: not available.

^g^FTM: female-to-male.

For professional and LGBT+ youth participants, internet safety and security issues were important, including the risk of LGBT+ young people being outed on the Web, challenges around how suicidality was safely managed in a Web-based context, and how LGBT+ young people can be specifically targeted for sexual exploitation. For these reasons, digital privacy and confidentiality appeared to be of fundamental importance. As described in focus group 2, with many LGBT+ young people adapting how they used technology for reasons of self-preservation and avoiding harassment:

It’s like you can get stalked on Snapchat now.

YP13, young person

Yeah.

YP17, young person

That’s the other thing. You can also put the ghost mode on so you can’t be followed.

YP13, young person

Yeah, because I’ve only chosen a few people that can see my location and [YP16 is] one of them.

YP17, young person

#### The Internet as a Resource and Tool

LGBT+ youth participants sought resources and material on the Web for reasons such as job searching, obtaining information, and to acquire health-related information (eg, *...how to put a condom on*). Some young people were aware of various organizations that had websites that they could access to support their mental health, and cited national examples such as ChildLine, MIND, and Stonewall. YouTube was also mentioned as a resource for mental health by several LGBT+ youth participants, for example:

...YouTube videos of like, because I have anxiety and my counsellor suggested doing something called, well, they suggested doing something called mindfulness, so like there are just some good videos on the internet of guided meditation and stuff which helps me through my anxiety.

YP20, young person, focus group 3

A common challenge mentioned by several professionals and LGBT+ youth participants was finding and accessing *good information* on the Web. As a professional highlighted:

...the issue with the internet is you have to know which of these multitude of sites is the one for you.

P4, professional, interview 3

Several participants shared examples of how the internet was an empowering tool or force for LGBT+ young people. For instance:

RUComing Out [a LGBT+ website], which shares celebrities but also real people’s experiences of coming out...And through that as a means to actually learn, discover, feel like there’s someone like them out there is really good.

P3, professional, interview 2

Professionals, in particular, highlighted that the internet was a valuable medium by which access to mental health services could be widened to potentially include *24/7* support. However, LGBT+ young people were acknowledged as being one of the *hard to reach groups*, and there would be some people who would not be comfortable accessing mental health services on the Web. Professionals also reinforced that any help on the Web should be blended with face-to-face therapy treatment options.

#### Digital and Personal Connections

Social media enabled LGBT+ young people to communicate freely with others and to be part of Web-based communities, something that was especially important to several youth participants:

I think with LGBT people it’s different, because I’d say we’re a more, what’s it called, a hated group by some people, and a bullied group so we would need more support.

YP20, young person, focus group 3

LGBT+ young people can also usefully connect with others who share their experiences and provide support online in ways that would be difficult for them to do in person:

...[The internet is] a really big support system...because not all, but most of the LGBT community have had hard times and they can all identify and relate with each other, so for the older [members of the]community they help out the younger [LGBT+ people] that are struggling.

YP19, young person, focus group 3

The internet was used for entertainment purposes (eg, to watch sports, shop online, and listen to music) as well as for other reasons. For instance, pornography was viewed as being both fun and for *sex education*, whereas selected forms of Web-based media provided helpful distraction (or company).

One LGBT+ young person said:

Yeah and I also use the internet, when I’m feeling low and depressed I also use it to distract myself. It’s a good coping mechanism, because it’s better than self-harming or getting too deep into my thoughts and having all these suicidal thoughts in my head, I can watch YouTube videos or I can go on Instagram or Tumblr or whatever...

YP20, young person, focus group 3

### Computer Games and Serious Gaming

The domain of *computer games and serious gaming* consisted of 3 themes: drawing on gaming experience; Rainbow SPARX as a resource for LGBT+ young people; and the look and feel of Rainbow SPARX.

#### Drawing on Gaming Experiences

Most of the LGBT+ youth participants reported playing a range of commercial games (eg, Rage, Skyrim, Mortal Kombat, and The Sims). They also outlined a variety of gameplay preferences: the game’s style of graphics; whether the game had a prologue; the amount of dialog that was used; and the degree of violence portrayed.

Some participants could see the therapeutic value of commercial games for mental health, as outlined by an LGBT+ young person:

I play a lot of the Lego games, just because they’re all the same controls and it’s easy and they’re so not stressful...I can just sit there and just zone out and just do anything, because they’re meant for like five-year-olds, so it’s fantastic.

YP17, young person, focus group 2

However, despite serious games being seen by some as valuable for those uncomfortable engaging with face-to-face therapy, challenges were identified for developing a serious game that would be acceptable to a range of LGBT+ young people. For instance, participants noted that such games would need to work for those of different abilities, levels of maturity, and stages of acceptance and understanding of their LGBT+ identities.

LGBT+ youth participants, in particular, highlighted that a mental health serious game should be available across a range of Web-based platforms (eg, on mobile phones, computers, and tablets). Moreover, a mobile serious game should not take up too much data storage.

#### The Rainbow Smart, Positive, Active, Realistic, X-Factor Program as a Resource for Lesbian, Gay, Bisexual, and Transgender Young People

After trialing module 1 of Rainbow SPARX during their interview or focus group, LGBT+ and professional participants recognized the purpose of the program (ie, it was intended to be a mental health tool for LGBT+ young people). However, several youth participants really wanted a more explicit focus on them and their particular needs, and some participants felt that Rainbow SPARX was inadequate as an LGBT+ resource:

...they don’t focus on the LGBT side of it, point blank, it is just mental health and I think if you were to do that it needs to be marketed as such. You can’t just change a few words around and have a slightly different message at the start and say “oh yeah it’s a completely different game for LGBT people.”

YP16, young person, focus group 2

Several LGBT+ youth participants also indicated that the language used in Rainbow SPARX was sometimes problematic:

...it mentions guys who like guys, like why don’t they just use the proper term? [ie, gay].

YP18, young person, focus group 2

In contrast, professionals appeared less concerned about whether the program had sufficient LGBT+ content and appeared more focused on whether Rainbow SPARX was too niche to be viable for a rollout:

I think there would be, given we’re talking about already a minority group. I think a minority of that minority group would find that platform quite attractive to use perhaps.

P1, professional, interview 1

Some LGBT+ youth participants also raised concerns about the avatar in Rainbow SPARX, in particular, the forced sex binary inherent in the program (ie, the user can only customize a male or female avatar with *no in-between* option). Although the avatars could already be customized in gender nonconforming ways (eg, the male avatar can have a *girl haircut* and wear luminous pink clothes), it was thought to be especially important to some youth participants to have nonbinary options for gender diverse users of the program.

##### Look and Feel of Rainbow SPARX

Several LGBT+ youth participants liked the *concept* of Rainbow SPARX, and they appeared to enjoy using the program:

Do you know, I’d definitely play that...

YP1, young person, focus group 1

Furthermore, the affective experience for some youth participants using the program was positive:

I really liked it. I would play it if it was released. I think it’s good, like it’s entertaining just as a game like if you were feeling stressed or bored or sad it would just take your mind off of it because it’s quite fun to do and then also I think it is good just the messages and stuff, I don’t know, it just cheered me up.

YP12, young person, interview 5

By contrast, a few young people reported that Rainbow SPARX was *patronizing*, and it would not be helpful for LGBT+ young people. In addition, participants also suggested that Rainbow SPARX was dated or needed refining in terms of the graphics:

...I don’t know exactly how it all plays out in the computer game world. But I think it’s pretty cutting edge...and that if you’re competing with that then that’s [Rainbow SPARX] going to look I think quite basic in comparison...

P3, professional, interview 2

They also highlighted issues in terms of speed:

It needs to be faster, it’s far too slow.

P1, professional, interview 1

And the controls:

I feel like maybe the actions and the freedom to move and what you could do on the game could be developed, like the movement was quite simple and stuff like just playing it and fighting the bad spirits...

YP12, young person, interview 5

There was also some discussion between LGBT+ youth participants about whether the spoken dialog in Rainbow SPARX was always understandable. There were times when young people seemed to struggle with the Māori (New Zealand indigenous language) phrases used in the program and a character’s accent. For example, the term takatāpui (a traditional Māori term meaning *intimate companion of the same sex*) was used in Rainbow SPARX, and this was a new word for participants in this study.

### The Well-Being Needs and Support of Lesbian, Gay, Bisexual, and Transgender Young People

This domain consisted of 2 themes: recognizing and acknowledging the LGBT+ youth experience and preferences and requirements for therapy and e-therapy.

#### Recognizing and Acknowledging the Experience of Lesbian, Gay, Bisexual, and Transgender Young People

LGBT+ youth participants described forms of mistreatment and other challenges that their heterosexual peers would not face. In particular, their family not accepting them because they are LGBT+ and the difficulties associated with accepting oneself, in part, because of internalized negativity:

...So when I was sprouting into the blossom that I am now, that part of the conflict came from not knowing what it [my own LGBT-specific identity] was. And the only context I’d ever heard of it being in-between was in like a promiscuous context like “oh that’s what people do if they have loose morals or anything"

YP17, young person, focus group 2

Furthermore, being transgender was described as being more stigmatized and resulting in increased mistreatment by a few participants, compared with those who were diverse in terms of their sexuality:

I think trans people are probably more at risk than LGB people, because it’s less normalized...So it’s like really overwhelming and stuff and I feel like, I don’t know, I feel like there’s more people on the internet preying on T people than LGB generally, because it’s easier to spot someone out and there’s more trans people who are like excluded from their families and stuff.

YP12, young person, interview 5

LGBT+ youth participants recognized that dual stigma could be an issue for LGBT+ young people, whereby they could be faced with the stigma associated with having mental health problems as well as the stigma linked to being LGBT+. Being isolated was also reported to be an issue, with this being *probably the worst thing.* To combat this, participants mentioned the value of LGBT+ characters on television and LGBT+ celebrity role models who were *completely unapologetic* about who they are (eg, Stephen Fry and Sir Ian McKellen), as a way to increase visibility.

Professionals also recognized that stigma creates barriers to LGBT+ young people getting help:

I think almost this group has double stigma because you would have the one around disclosing that you might have some mental health difficulties, and then the additional stigma of being, lesbian, gay, or transsexual on top of that. So there would be quite a lot of barriers for you to come out and start talking about how you feel about things.

P2, professional, interview 1

Developing and maintaining LGBT+ communities on the Web was especially important for youth participants. This allowed them to date other LGBT+ young people and engage in leisure activities with other LGBT+ youth (eg, an online friendship had the potential to lead to face-to-face activities), and it provided a sense of LGBT+ community and belonging.

#### Preferences and Requirements for Therapy and Electronic Therapy

Participants reinforced that CD-ROMs and websites were viewed as outdated means by which to offer an intervention. Access issues would also need to be taken into account; for instance, Wi-Fi is not always freely available to young people, so it was recommended that Web-based e-therapies have a downloadable option. Serious games presented their own set of problems in terms of the need to move in line with expectations based on commercial games:

I think what you need to think about as well is the life span of the game. So gaming in general will have updates every single year because there is quite a lot of competition.

P2, professional, interview 1

Some professional participants were particularly concerned with the effectiveness of an e-therapy. RCTs were cited as a means to provide the evidence required to demonstrate that an e-therapy was effective, and so the focus was on demonstrating effectiveness at a population level although their limitations were recognized:

I suppose it’s a question about a rigorous evaluation, you know, like an RCT, versus something which is, I don’t know, a sort of user-experience evaluation where we’re not going to randomize people because if people want to use it then they should be able to use it, you know, rather than saying well, fine but we’re going to randomize you to a waitlist control now.

P4, professional, interview 3

In contrast, LGBT+ youth participants seemed to evaluate cCBT interventions (such as Rainbow SPARX), more generally, in terms of their ideas about the perceived usefulness of the CBT content:

...where it [Rainbow SPARX] said things that you can do or ways you can think to change how you feel. Which is a concept that works but sometimes it’s really not helpful to hear. And it’s obviously not that exciting.

YP4, young person, focus group 1

Professional participants were especially concerned that any e-therapy has sufficient moderation and guidance to ensure risk is managed effectively. There was a consensus among professionals that an e-therapy should be provided in a blended way so that LGBT+ young people always had the option of face-to-face therapy, if required.

Among LGBT+ youth participants, there was some debate about how explicit therapy should be in a serious game, with some suggesting that this should almost be achieved by stealth:

So do you think there’s a way that you could innovate the game to where it’s not therapy talk...it’s just, it’s a way of not thinking that it’s therapy talk.

YP4, young person, focus group 1

Commissioning e-therapies on the NHS requires certain criterion be met, specifically around effectiveness and safety, as explained by a commissioner:

...in our specification we will have a standard sentence around that it has to be evidence-based. And you sort of hang all sorts of things off that really. So in the procurement process we went through they had to show us evidence of how that particular sort of online service was going to work and that it was going to be safe.

P2, professional, interview 1

However, commissioning was acknowledged by some professionals to involve further challenges; in that, for an e-therapy to be *attractive* for a health service commissioning body, it would need to be relevant to *a huge group* of young people for each commissioning body to support its implementation.

### Questionnaire Responses

Of 21 LGBT+ youth participants, 18 (18/21, 86%) indicated that they had felt down or low in the past (based on the single question: *have you ever suffered from feeling down or low for more than a few days in a row?*). The remaining 3 participants (YP8, YP9, and YP11) reported having not previously suffered from feeling down or low. Most professionals said that they would recommend Rainbow SPARX to an LGBT+ young person who was *feeling down* (4/6, 67%), whereas only 38% (8/21) of the youth participants reported that they would use Rainbow SPARX if *feeling down* themselves. Most participants provided comments about why they would or would not use (or recommend) the program (see Table 3).

**Table 3:** Participants’ written responses.

Would recommend use of Rainbow SPARX^a^		Comments
**Professionals**		
	Yes (n=4)	"I would recommend that they try it, but would follow up to see if they felt their needs were met, or if further assistance was required." [P1, professional, interview 1] "Yes in principle, but would like to see final version [ie, all 7 modules] first before recommending." [P2, professional, interview 1] "As part of or accompanying face-to-face intervention." [P3, professional, interview 2] I think it’s a great, accessible self help tool" and "Lovely game, very useful, accessible." [P5, professional]^b^
	Possibly (n=1)	"Possibly – depends on whether they feel the internet intervention would be helpful. Many prefer face to face interventions." [P4, professional, interview 3]
	No (n=1)	"I think they need someone to talk to face to face" and "It will provide extra support." [P6, Professional]^b^
**Young people**		
	Yes (n=8)^c^	"I enjoyed it." [YP12, individual interview 5] "It’s awfully good." [YP17, focus group 2] "For anxiety and when my mood is especially low." [YP 19, focus group 3] "For anxiety." [YP20, focus group 3] "I think it could help up to a poin[t] [sic]." [YP21, focus group 3]
	No (n=13)^d^	"I have nothing to add." [YP2, focus group 1] "Would rather develop skills when better." [YP4, focus group 1] "Bit too basic and CBT for my liking." [YP6, focus group 1] "I’d see my care coirdinator [sic] instead." [YP7, focus group 1] "I don’t require a game to make me feel better I have [P5, Professional – LGBT+ stakeholder] and [P6, Professional – Mental health]." [YP8, focus group 1] "I don’t think it would help me." [YP13, focus group 2] "More of a distraction than how to solve a problem." [YP14, focus group 2] "Outdated system/terms." [YP15, focus group 2] "No thanks." [YP16, focus group 2] "Skims over topic at hand." [YP18, focus group 2]

^a^SPARX: Smart, Positive, Active, Realistic, X-factor thoughts.

^b^The same participant completed 2 surveys.

^c^Participants YP1, YP3, and YP11 did not provide a written comment, for reasons unknown to the authors.

^d^Participants YP5, YP9, and YP10 did not provide a written comment, for reasons unknown to the authors.

## Discussion

### Principal Findings

This study sought to explore 2 main research objectives. First, to explore how and why LGBT+ young people use the internet to support their mental health. This is important to consider to minimize the risks of developing interventions that do not address LGBT+ users’ needs or do not fit-in well with how LGBT+ young people use the internet. Notably, rather than accessing existing e-therapies developed for the general youth population, young people in our study created personal pathways to use the internet for enhancing their mental health (eg, by locating resources on mindfulness via YouTube). Their apparent lack of knowledge about e-therapies, combined with the challenges associated with finding and accessing *good information on the Web*, suggests that even if LGBT+ e-therapies were made available, they would be difficult for LGBT+ young people to locate. Therefore, it is not simply the development of e-therapies and demonstrating their effectiveness that needs consideration but also their dissemination and ensuring they are adequately and appropriately supported in the real world [31]. Another issue of relevance for LGBT+ young people was cyberbullying and stalking. Professional participants, in particular, were concerned about the safety and the personal security of LGBT+ young people on the Web and described concerns about the risk of Web-based sexual exploitation. Hence, e-therapies for LGBT+ young people will need to carefully consider these issues in their development, evaluation, and implementation.

The second objective of this study was to elucidate whether LGBT+ young people and professionals consider an e-therapy, such is Rainbow SPARX, as a useful tool to assist in supporting the mental health of LGBT+ young people. Most of the professionals indicated that they would recommend the program to an LGBT+ young person who was *feeling down*, whereas only 8 of the 21 LGBT+ young people indicated that they would use Rainbow SPARX in this context. In part, usefulness will be about effectiveness, but another important factor to consider is acceptability. A key means by which to enhance acceptability is to use codesign or coproduction processes. This study found diverse attitudes to gaming preferences as well as different opinions about the appeal of Rainbow SPARX and factors related to LGBT+ identities. It will be important that future e-therapy codesign approaches for this population pay special attention to ensuring that a range of LGBT+ individuals take part in these processes, in particular, transgender young people [32]. Rainbow SPARX was originally developed using codesign methods [6]. However, these processes occurred some years ago and in a different cultural context (that of New Zealand). As LGBT+ young people are coming out at increasingly early ages [33], they are now even more likely to require content that is specific to their experiences as LGBT+ individuals, especially because the majority of existing LGBT+ services are provided for adults in large urban centers. Another challenge relates to the use of serious games as a therapeutic medium. In particular, the pressure to remain as up-to-date as possible relative to commercial games, as these games appear to be the base of comparison from which young people draw on when critiquing an e-therapy delivered in a game-like format.

### Comparisons With Prior Research

A considerable body of research has been published, which reinforces that LGBT+ young people are an *at-risk* population in terms of their mental health. However, to date, very few studies have focused on the means by which to address the mental health problems that arise from hostile environments [4] from which LGBT+ young people may not be able to escape. For instance, in a recent systematic review of empirically based psychological treatments conducted with LGBT+ young people, Hobaica et al identified only 8 such interventions [20]. Of the 8 interventions, 3 were Web-based, specifically: Rainbow SPARX; *Queer Sex Education* (an inclusive sex education intervention); and a Web-based drug abuse prevention intervention [20]. Another similar systematic review, conducted by Van Der Pol-Harney and McAloon [10], highlighted only one e-therapy for mental health problems (ie, Rainbow SPARX). However, the e-therapy field is rapidly evolving, and the developers of AFFIRM (a group-based CBT intervention for LGBT+ young people, cited in both reviews) [34] have highlighted their intention to expand their program. In particular, it is hoped that, in the future, AFFIRM can be delivered in Web-based format [35]. We identified another e-therapy intervention in the field of LGBT+ mental health called TODAY. This tool is a mobile phone intervention for the treatment of anxiety and depression and was developed in the United States. TODAY has been subjected to usability testing [36]. However, to date, it has been exclusively evaluated to inform further development among gay-identified young men (aged 18-20 years) [36]. Given that Web-based psychosocial tools are valued by LGBT+ young people [16] and that they can be made accessible to socially and geographically isolated individuals [5], effective and acceptable e-therapies should be made available to LGBT+ young people in the United Kingdom and elsewhere. It is probable that funders of e-therapies may argue that the target population is not large enough to justify the investment. But given the extent of the problems [1,3], that LGBT+ young people are coming out earlier [33], and that therapy access issues have previously been identified [9], early intervention is warranted. Rollout is also likely to be more cost-effective if LGBT+ resources can be funded across several clinical commissioning groups or public health departments.

For e-therapies to be used meaningfully by LGBT+ young people, they will need to include relevant content. Although e-therapies are demonstrably important tools in addressing mental health problems, the vast majority are designed for a general population [37]. But these *mainstream* tools do not address the needs of LGBT+ individuals. For instance, Rozbroj et al reviewed Web- and app-based interventions for the prevention and treatment of depression and anxiety in relation to the degree to which they would meet the needs of lesbians and gay men (of note, the review was not inclusive of bisexual or transgender individuals). They found that the tools largely neglected core issues for lesbians and gay men [37], such that more than half (14 of 24 interventions) contained instances that assumed or suggested the user was heterosexual. Moreover, only 1 intervention explicitly addressed homonegativity, and only 1 tool referred to same-gender relationships [37].

Given the findings from this study, which indicated that a minority of LGBT+ young people who participated in this study would use Rainbow SPARX, but that discussions revealed others might if the content and format were improved, this research suggests that adapting existing resources designed for LGBT+ youth can be a worthwhile endeavor. Nonetheless, whether new LGBT-specific e-therapies are created or whether they are modified from existing interventions, to ensure they are up-to-date, all e-therapies need to be more rapidly tested and implemented [38].

### Implications

In this exploratory study, we have highlighted that LGBT+ young people are interested in mental health support via the internet and that e-therapies should be tailored for LGBT+ young people and their cultural context. We have further identified that the needs and preferences of LGBT+ young people are diverse and, in some cases, polarized. For example, the affective experience with Rainbow SPARX included those LGBT+ young people reporting positive emotions associated with the program and others, in contrast, who felt patronized by the language used within the game. It may not be as simple as developing 1 tool or approach that suits all LGBT+ young people. However, there could be future scope to develop multiple *layers* within the same e-therapy (game-based or otherwise) that are tailored to appeal to different developmental or maturity levels. This is an approach we intend to explore in the future. An approach that is personalized in this way could allow for the customization of language and design that is deemed acceptable for a wide range of LGBT+ young people. This would be useful because choice and control are obviously important considerations in the design of serious games for adolescents [39]. Furthermore, this study has shown that timeliness or recency of approach is important because internet intervention’s date and the expectations of LGBT+ young people appear high.

### Limitations

This is a small-scale exploratory study, and as is not uncommon in research conducted in the area of LGBT+ mental health, recruitment had its challenges [27]. The study included 4 LGBT+ young people aged older than 19 years (whereas Rainbow SPARX was designed for young people up to this age) with the oldest participant being aged 22 years. However, these participants were established members of their youth group, they were keen to take part in a focus group, and they provided useful insights, as young people just outside of the initial target age range.

This study’s sample was not representative of all LGBT+ young people in the United Kingdom or the professionals working with them. Furthermore, not all LGBT+ youth organizations in the United Kingdom are likely to have been contacted, especially those organizations that do not have a Web presence outside of London, Cardiff, Manchester, Edinburgh, and Belfast. Some young people from the organizations contacted may not have heard about this study. Regardless of our attempts to recruit them, no parents or guardians of LGBT+ young people took part in this study. Despite this, we had a range of LGBT+ young people and professionals taking part in this study, including young people questioning their sexuality and those who were nongender binary. We had a strong sense that no new concepts or ideas were raised on the conclusion of focus group 3. Transcripts were not returned to LGBT+ youth or professional participants for comment or for participants’ feedback on the findings. No field notes were taken.

When using Rainbow SPARX in focus groups, only 1 young person played the game at a time while others watched and commented. However, Rainbow SPARX was designed for use in a single player format. It is, therefore, possible that using the program in this manner influenced how much participants were able to engage with this e-therapy. Moreover, participants knew that ML led the development of Rainbow SPARX, and as such, young people may have felt reserved in relation to expressing criticisms of the program. To attempt to remedy this, every participant was explicitly asked to comment on what they did not like about Rainbow SPARX. Nonetheless, it is possible that the results are skewed toward a more positive view of the program. In focus groups 1 and 3, the professionals responsible for these youth groups chose to attend these focus groups. The inclusion of these professionals in 2 youth focus groups is a limitation, as their presence may have influenced what young people said. However, professionals’ data from the focus groups were not included in analyses, and our impression is that having these professionals present made young people feel at ease.

### Conclusions

LGBT+ young people frequently experience stigma and isolation, and they also have high mental health needs. The internet is an important source of information and support for these young people, and e-therapies appear particularly valuable for this unique population. In this study, the first where Rainbow SPARX was used outside of New Zealand, LGBT+ young people emphasized that e-therapies must be appealing, up-to-date and inclusive of LGBT-specific content. Professional participants reinforced the need for proof of efficacy and that an e-therapy appeals to a sizeable proportion of a population. LGBT-specific e-therapies, such as Rainbow SPARX, show promise, but only those that are tested sufficiently should be made available to support the mental health of LGBT+ young people. To reduce costs and increase access, these tested interventions should be considered for implementation by commissioners across wide geographical areas.

## Appendix

Multimedia Appendix 1 Focus group guide.

## Abbreviations

cCBT: computerized cognitive behavioral therapy
e-therapy: electronic therapy
LGBT: lesbian, gay, bisexual, and transgender
LGBT+: LGBT and other people diverse in terms of their sexuality and gender
NHS: National Health Service
RCT: randomized controlled trial
SPARX: Smart, Positive, Active, Realistic, X-factor thoughts
