# Supplementary material for: How LGBT+ Young People Use the Internet in Relation to Their Mental Health and Envisage the Use of e-Therapy: Exploratory Study
Source: JMIR Serious Games. 2018 Dec 21;6(4):e11249. doi: 10.2196/11249 (PMC6320432; doi:10.2196/11249)
Supplement: Multimedia Appendix 1 [file games_v6i4e11249_app1.pdf]

Appendix 1. Focus group guide

Session plan

One or two researchers from the project team will facilitate each focus group session (Open University Facilitator/OUf) – LGBT+ group facilitators (e.g. youth workers in charge of the LGBT+ youth group) may select to also participate in a focus group (where they are in charge of this group).

Before the group assembles

OUf - Test the recording equipment to make sure it is working and that the sound is recording at an acceptable level. Check Rainbow SPARX program is working on OUF's laptop.

OUf - Ensure room lay-out appropriate and all paperwork ready - notes, name badges, demographics form, and participant consent forms (if not previously returned by post/email).

OUf – Ensure snacks and soft drinks are available.

Preparing to start the session

OUf - Offer refreshments.

OUf - Ensure all consent forms appropriately completed. Remind participants regarding confidentiality of the session and discuss group rules. Remind participants that this is audio-recorded so that this can be transcribed.

OUf - Ask members of the group to introduce themselves giving their names and correct pronouns. Also as an ice-breaker, ask young people if they could never access the internet ever again what would they miss the most (e.g. YouTube, Facebook etc).

Introduction to the session

OUf - Reiterate the purpose of the focus group. Remind participants the focus is on understanding how the internet can be used to support 'rainbow' or LGBT+ youth wellbeing. State that the internet can include many things, including what young people access on a smart phone, online games, and anything on the web.

Discussion 1: Internet uses

Q1. What are the main reasons why young people use the internet?
(Prompts - For what information, tools, or resources? What is your preferred format (i.e. laptop, phones, PC etc) for using the internet? How can the internet help rainbow young people connect with other people?)

Q2. Do you use the internet when stressed or low?

Q3. How can the internet be helpful when someone like you is stressed or low?
(Prompts – If you had mental health issues would you use the internet? How would you do this? What online sites or resources do young people find useful or helpful for mental health issues? What online sites or resources do young people not find useful or helpful for mental health issues?)

Discussion 2: Rainbow SPARX

Ask group participants the following questions whilst they are trying out Rainbow SPARX on the OUf's laptop (i.e. participants can take turns trying it out, and others can observe, comment, and ask questions).

Q4. Do you play computer games? If yes, what games do you play at the moment?

Q5. What do you think of Rainbow SPARX?
(Prompts – What do you like about it? What don't you like about it?)

Q6. Rainbow SPARX was developed in New Zealand – does it need adapting to be used in the United Kingdom?
(Prompts – What changes are needed/essential? What changes would be helpful, but not essential?)

Q7.What might stop someone like you using Rainbow SPARX if it were made available in the United Kingdom?
(Prompts – What would make it easy to access Rainbow SPARX? What would make it hard to access Rainbow SPARX? What is the best format for SPARX – phone, PC, laptop?)

Q8. Would you recommend Rainbow SPARX to someone like you if they were feeling stressed and low? Why, or why not?

Ending the session

OUf - summarize the discussions. Remind participants a summary of findings will be available if requested (and email is provided) and remind them of details of

contacts for further guidance on wellbeing if needed (on information sheet). Ask participants to complete the demographics form.

Provide participants with their vouchers and thank them for their participation.
